# Auto detection and segmentation of daily living activities during a Timed Up and Go task in people with Parkinson’s disease using multiple inertial sensors

**DOI:** 10.1186/s12984-017-0241-2

**Published:** 2017-04-07

**Authors:** Hung Nguyen, Karina Lebel, Patrick Boissy, Sarah Bogard, Etienne Goubault, Christian Duval

**Affiliations:** 1 Département des Sciences de l’activité Physique, Université du Québec àMontréal, 141 Avenue du Président -Kennedy, Montréal, Québec Canada; 2 Centre de Recherche de l’Institut Universitaire de Gériatrie de Montréal, Montréal, Québec Canada; 3 Faculty of Medicine and Health Sciences, Department of Surgery, Université de Sherbrooke, Sherbrooke, Québec Canada; 4 Research Center on Aging, Sherbrooke, Québec Canada; 5 Interdisciplinary Institute for Technological Innovation (3IT), Université de Sherbrooke, Sherbrooke, Québec Canada

**Keywords:** Accelerometer, Movement disorders, Sitting, Turning, Walking, Parkinson’s disease, Activity detection, Inertial measurement unit

## Abstract

**Background:**

Wearable sensors have the potential to provide clinicians with access to motor performance of people with movement disorder as they undergo intervention. However, sensor data often have to be manually classified and segmented before they can be processed into clinical metrics. This process can be time consuming. We recently proposed detection and segmentation algorithms based on peak detection using Inertial Measurement Units (IMUs) to automatically identify and isolate common activities during daily living such as *standing up*, *walking*, *turning*, and *sitting down*. These algorithms were developed using a homogenous population of healthy older adults. The aim of this study was to investigate the transferability of these algorithms in people with Parkinson’s disease (PD).

**Methods:**

A modified Timed Up And Go task was used since it is comprised of these activities, all performed in a continuous fashion. Twelve older adults diagnosed with early PD (Hoehn & Yahr ≤ 2) were recruited for the study and performed three trials of a 10 and 5-m TUG during OFF state. They were outfitted with 17 IMUs covering each body segment. Raw data from IMUs were detrended, normalized and filtered to reveal kinematics peaks that corresponded to different activities. Segmentation was accomplished by identifying the first minimum or maximum to the right and the left of these peaks. Segmentation times were compared to results from two examiners who visually segmented the activities. Specificity and sensitivity were used to evaluate the accuracy of the detection algorithms.

**Results:**

Using the same IMUs and algorithms developed in the previous study, we were able to detect these activities with 97.6% sensitivity and 92.7% specificity (*n* = 432) in PD population. However, with modifications to the IMUs selection, we were able to detect these activities with 100% accuracy. Similarly, applying the same segmentation to PD population, we were able to isolate these activities within ~500 *ms* of the visual segmentation. Re-optimizing the filtering frequencies, we were able to reduce this difference to ~400 *ms*.

**Conclusions:**

This study demonstrates the agility and transferability of using a system of IMUs to accurately detect and segment activities in daily living in people with movement disorders.

## Background

People suffering from movement disorders often experience limited mobility, which could lead to loss of independence and a decrease in the quality of life [1, 2]. Recently, much attention has been given to the use of body-worn sensors to monitor mobility [3–6] in an effort to improve patient care through real-time feedback of rehabilitation [7, 8] and pharmaceutical intervention [9], particularly in patients with Parkinson’s diseases (PD). These sensors are ubiquitous in the detection of physical activities such as *walking*, *sitting* and *standing* during the course of daily living in clinical setting [10–13] as well as in free-living environment [14–18].The automation of the detection and segmentation of these activities could precipitate the analysis of the quality of the movement, which will provide clinicians with real-time motor function behavior to adapt their treatment strategies to improve patient cares and ultimately increase the quality of life for people with movement disorders.

Inertial Measurement Unit (IMU), which are comprised of a 3D accelerometer, a gyroscope and a magnetometer, is widely used in many applications. These sensors have the potential to provide continuous remote monitoring in natural environments, and therefore, are more practical to deploy than laboratory-based optical motion capture systems. More than ever, one of the emerging uses for IMUs is to detect daily living activities and assess the quality of the movement during these activities. For example, a system combining inertial and barometric sensors on different anatomical locations was used to detect activities such as drinking and writing [10]. Postural transitions especially during *sit-to-stand* and *stand-to-sit* have also been detected with high accuracy using a single chest mounted gyroscope [19] and a tri-axial accelerometer [20, 21] in clinical settings to evaluate mobility. However, the emphasis of these types of system has been on the detection of activity. In addition, the scope of these postural transition detections has been limited to static transition and the range of the activity that can be detected is limited by the amount of sensor information available. Many studies have also focused on the use of sensors to characterize the quality of the movement in people with PD. Zijlstra [22] showed that patients with PD displayed a lower angular velocity during the extension phase of standing up using an inertial sensor on the hip. Similarly, parameters such as trunk angle [23], freezing of gait, [24–26]and gait parameters [27–29] (stride time, cadence, range of motion, etc.) have been shown to change significantly in people with PD when compared to healthy older adults during common daily living activities. Furthermore, turning step [30] and speed [31] have been extracted from inertial sensors to evaluate the motor quality of people with PD during turning task.

**Fig. 1:**
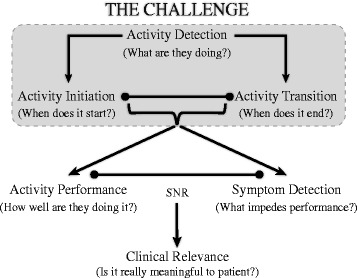
The challenge of detecting, segmenting and analyzing ADL in patients with movement disorder. The focus of this study is on the activity detection, indicated in *gray*

However, for remote monitoring of patients in their natural environment using IMUs to be efficient, there need to be a systematic approach for the development of such tool. First, one must be able to detect what the person is doing (e.g., *walking*, *sitting*, etc.), as well as detect transitions between tasks (e.g., initiation of gait). Also, within those segments, a proper detection of symptoms can be done (e.g., tremor, bradykinesia, freezing, etc.). To be clinically relevant, it must be determined whether the detected symptom had an effect on motor performance. This is evaluated using a signal-to-noise approach [32] where the signal is the voluntary movement and the noise is the symptom detected. If the signal-to-noise is high, then the symptom is irrelevant to the performance of the person tested. This process is illustrated in Fig. 1. The present study focuses on the *Activity Detection* highlighted in gray.

We recently proposed detection and segmentation algorithms based on peak detection of IMUs data to automatically isolate common activities in daily living in healthy older adults [14, 15, 33]. Using multiple IMUs positioned on different limb segments on the body, we were able to accurately segment and detect activities of daily living in a homogenous healthy aging population using kinematics and orientation data obtained from these IMUs. However, people with PD often exhibited altered gaits, reduced strength, and limited mobility due to neurological damages caused by the disease, which could affect the generalizability and application of the detection and segmentation algorithms developed in healthy older adults. Specifically, tremor, rigidity, and bradykinesia can manifest in patients with PD, which can also cause difficulties performing simple movements (e.g. standing up from a chair). These conditions can magnify the challenges these patients face in simple tasks such as *turning*, where they adopt “en block” movement to overcome these physical limitations. PD patients can also develop abnormal gait during walking such as shuffling feet and freezing of gait. The physical limitations of PD can alter the sensor markers used to accurately detect and segment activities common in daily living. Further compounding the challenges, PD affects each patient very differently, thus making it difficult to develop a universal algorithm based on IMUs to capture and segment these activities. The aim of this study was to investigate the transferability of these algorithms in detecting and segmenting these common activities in people with PD during OFF state. Similar to previous study, we used a modified Timed Up And Go (TUG) task because it contains four common activities such as *standing up*, *walking*, *turning* and *sitting down* performed in a continuous fashion.

## Methods

### Participants

Twelve community dwelling older adults (4 females; 67.8 ± 10.4 years old, height = 1.66 ± 0.04 m, weight = 54.0 ± 7.8 kg, BMI = 19.8 ± 3.6 kg/m^2^; 8 males, 66.6 ± 3.6 years old, height = 1.77 ± 0.04 m weight = 79.9 ± 17.3 kg, BMI = 25.7 ± 6.1 kg/m^2^) who are diagnosed with early stages PD were recruited for the study. Patients were recruited through the Centre de Recherche de l’Institut Universitaire de Gériatrie de Montréal (CRIUGM) in collaboration with Quebec Parkinson Network(QPN). Participants were screened for cognitive deficits using the Montreal Cognitive Assessment (MOCA) test (mean = 27.7 ± std = 2.3) [34]. None of the participants exhibited any physical limitations or pain that could affect their ability to perform the task. The Nottingham Activity of Daily Living Scale was used to ensure that the participants were independent in their living environments. All participants involved in the study were rated less than or equal to 2 on the Hoehn and Yahr [35] scale (1.4 ± 0.8) to form a homogeneous motor symptom of early PD and avoid the more severe motor symptoms during the later state of PD. Other sub-scores from the motor examination portion of the MDS-UPDRS assessment such as arising from the chair, gait and posture were also recorded. While only early PD participants were recruited, some participants exhibited motor symptoms such as rigidity, tremor, and bradykinesia. The physical and motor characteristics of the participants are summarized in Table 1. The institutional research ethics review board of the CRIUGM approved this research and each participant read and signed an informed consent form.

**Table 1:** Motor symptoms and mobility characteristic of the participants

				Motor symptoms				MDS-UPDRS motor examination sub-score				
Part.	Age	Height (cm)	Year	First symptom	Rigidity	Tremor	Brady.	Arising from chair	Hoehn & Yahr	Gait	Posture	MOCA
1	61	171	8	Tremor		✓		0	2.0	0	0	27
2	64	183	7	N/A		✓		0	2.0	0	0	30
3	79	163	9	Tremor	✓		✓	1	1.5	1	0	23
4	68	173	3	N/A		✓		1	1.5	0	0	26
5	73	178	15	Rigidity				0	1.0	0	0	24
6	62	173	3	Tremor				0	1.0	0	2	25
7	67	180	3	Rigidity				0	1.0	0	0	27
8	74	163	5	Tremor				1	1.0	0	0	29
9	70	178	4	N/A	✓			1	2.0	0	0	26
10	65	178	3	N/A	✓			0	1	0	0	29
11	64	172	0	N/A	✓			0	2.0	0	0	30
12	57	166	4	Brady.				0	2.0	0	1	26
Mean ± Std	**67 ± 6**	173 ± 7	5 ± 4					0.33. ± 0.49	1.4 ± 0.8	0.08 ± 0.29	0.18 ± 0.40	27 ± 2

Sub-scores from the motor examination portion of the MDS –UPDRS and the motor symptom of PD participants are tabulated. Cognitive assessment was evaluated using the Montreal Cognitive Assessment test (MOCA). MOCA scores are scaled out of a possible 30. Year indicates how long participants have been diagnosed with PD

### Experiment protocol

**Fig. 2:**
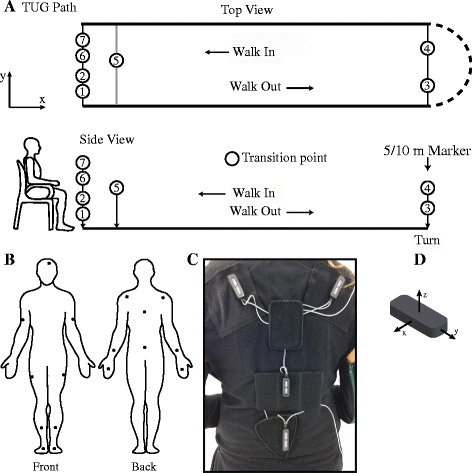
Schematic of the TUG task and motion capture system based on IMU. **a** Spatial schematic of a TUG path and different transition points. Seven transitions were identified among the activities performed during a TUG. These transitions are: 1) *sit-to-stand* 2) *stand-to-walk out* 3) *walk out-to-turn* 4) *turn-to-walk in* 5) *walk in-to-turn* 6) *turn-to-stand* 7) *stand-to-sit*. **b** Diagram of the 17 IMUs and their locations on the suit. **c** A close-up view of the IMU on the shoulders, trunk, and hip. **d** Using the right-hand Cartesian coordinate system, the y-axis is aligned along the length of the IMU while the x-axis is aligned along the width of the IMU. Most IMU were positioned on the body with the y-axis aligned along the limb segment, except for the IMU on the head, where the x-axis was aligned with the rotation of the head

Participant were tested in the morning during their OFF state or at least 10 h after their last medication. Participants performed two TUG tasks, one having length of 10 m, the other 5 m. Participants performed three trials of each TUG task. Data recording started with participants in a standing position to initialize the IMUs. Participants then sat down in a armed-chair to perform the task. Participants were asked to stand up without using their arms, walk to a marker on the floor (5 m and 10 m), turn around, walk back to the chair and finally sit down (Fig. 2a). Participants were asked to perform these tasks at their own pace and no instructions were given on how sit, walk, or turn.

Participants performed these TUG tasks while wearing an IGS-180 motion capture suit (Synertial UK Ltd, Brighton, UK). The IGS-180 (Fig. 2b-d) is equipped with 17 IMU modules (OS3D, Inertial Lab, VA, USA) positioned on each limb segment in order to capture the full-body 3D movement. Each IMU module is comprised of 3-axis linear acceleration (accelerometer), angular velocity (gyroscope) and magnetic north heading (magnetometer). Raw data (acceleration, angular velocity) and 3D orientation (estimated from a proprietary fusion algorithm developed by Inertial Lab) from each IMU were acquired at 60 Hz. Since there was no *a priori* expectation as to which IMUs were suitable markers for detection and segmentation, all 17 IMUs were active during the recording. This allowed us to identify the best set of IMU to detect and segment movement during the TUG.

### Detection and segmentation

**Fig. 3:**
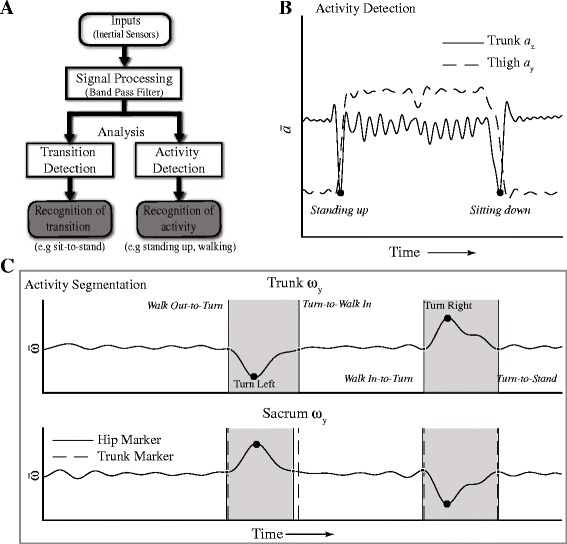
Summary of global detection and segmentation algorithm. **a** Global workflow of the algorithm to detect the activities and transitions between activities using a motion capture system based on IMU. **b** Activities were detected by identifying peaks in selected IMUs that corresponded to different activities. $\overline{\alpha}$ and $\overline{\omega}$ denotes the normalized acceleration and angular velocity of the IMU. Multiple IMUs were used to provide complementary information that yielded more robust and accurate detection of activities. Here *standing up* was detected using the trunk *a*_z_ and the time derivative of the thigh *a*_y_ (${\overset{.}{\alpha}}_y$ >0). Similarly, *sitting down* was detected using the same IMU with (${\overset{.}{\alpha}}_y$ <0). **c** Segmentation was achieved by identifying the minimum/maximum to the *left/right* of the activity peaks. Multiple IMUs could be used to detect the same transition. The average time marked by these IMUs were used to estimate the beginning/ending of each activity. Here the sacrum and trunk angular velocities (ω_y_) were used to estimate the transition that involved *turning*

The algorithms developed to detect and segment activities during TUG are described in detail elsewhere [33] (Fig. 3a). In brief, selected IMUs were identified and processed using a band pass filter at optimal frequencies to reveal kinematics peaks that corresponded to different activities. For activity detection, multiple IMUs were be used to provide complementary sensor information to distinctively identify different activities. For example, the peak of the trunk acceleration (*a*_z_) could indicate both *sitting down* and *standing up*, however; augmenting that information with the derivative of the acceleration (${\overset{.}{\alpha}}_y$), we were able to differentiate between these two activities (Fig. 3b). For most IMUs, the directional axis refers to the local reference frame of the IMU with the y-axis aligned along the limb segment, except for the head, where the x-axis was aligned with the axial rotation of the head. Once these activities were detected, the transitions (beginning and end) of these activities were identified by locating the minimum or maximum to the left or right of the activity peaks. This process essentially calculated the width or duration of the activity. In more dynamics transitions, multiple IMUs were used to estimate the transition point by averaging the times marked by these IMUs (Fig. 3c). This is due to the asynchronous movement of the limb segments and the variability in how participants strategized their movements to transition from one activity to the next.

Two independent examiners also segmented the activities during the TUG using the visual full body avatar generated from IGS-180 motion capture software to measure the accuracy of the segmentation algorithm. These two examiners were given instruction on how to visually mark the beginning and end of the different activities during a TUG, but no specific markers were imposed to prevent bias on the algorithm. Examiners were instructed to mark the transition from sit-to-stand by identify the upward movement of the body from the sitting position, but no specific body movements was stipulated. Participants were instructed to perform the task at their own volition to mimic their natural movement; therefore, a general guideline was needed to account for the variability in how participants transition from one activity to the next. For example, during the transition from stand-to-walk, some participants made discretize movements from stand up to walking while some transitioned to walking immediately from sitting position. Thus the examiners must use their judgments to identify these differences among participants. Given the general guideline, the intra-rater reliability between the examiners was excellent (ICC = 0.99).

The algorithms that were previously developed based on a population of healthy older adults were first used to detect and segment activities in PD participants to determine their accuracy and transferability. Subsequently, the algorithms were re-optimized to adapt to PD participants and to improve their accuracy. They were modified in participants with PD using the data from the 10 m trials; however, the algorithms were applied to the 5 m trials without any modifications or re-optimization. While this does not addressed the transferability of the algorithms to all patients with PD, regardless of stage or type of disease, it highlights the need for adapting the number of IMUs and algorithms to the tested population.

IMU and signal optimization were performed to improve the accuracy of the detection and segmentation of these activities that might have been affected by the motor differences between healthy older adults and people with PD. Additional IMUs or sensor signals were needed to develop a more robust redundant system that was capable of capturing the natural movement of the participants as they performed these tasks in an unimpeded and continuous motion.

Sensitivity and specificity [36] were used to evaluate the performance of the algorithm to detect the activities performed during a TUG in comparison with the visually segmented ground truth. Sensitivity measures the proportion of actual positive activities detected (true positive) while specificity measures the proportion of the negative activities that were detected (true negative). The timestamp differences (ΔT = T_Sensor_–T_Visual_) between the transition times segmented visually by two examiners and the IMUs were used to evaluate the performance of the algorithm across twelve participants at each transition.

## Results

### Activities detection

Using the same detection algorithms that were developed using a population of healthy adults to PD population, we analyzed 12 participants performing 3 trials of a 5 m and 10 m TUG that yielded 432 (12 participants x 6 activities x 3 trials x 2 tasks) instances of activities such as *standing up*, *sitting down*, *walking*, and *turning*. The results show that during *standing up*, the detection sensitivity was 100% for both the 10 m and 5 m TUG (*n* = 72) while specificity was 94.7% and 97.3%, respectively. Similarly, during *sitting down,* the sensitivity was 100% for the 10 m and 5 m TUG and specificity was 97.3% and 92.3%, respectively (*n* = 36). During the 10 m TUG, *walking* was detected with sensitivity of 91.6% while specificity was 87.5% (*n* = 72). In the 5 m TUG, *walking* sensitivity was 88.9% and specificity was 72.2% (*n* = 7*2*). *Turning* was detected with 100% sensitivity and specificity during both TUG tasks (*n* = 144).

### Modifications for patients with PD

**Fig. 4:**
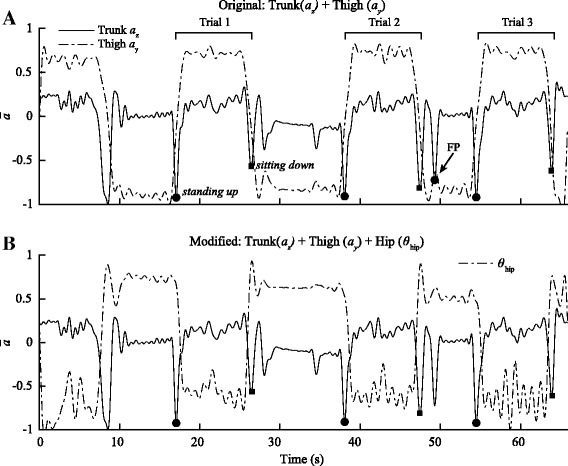
Comparison between the original and modified algorithms for the *standing up* and *sitting down*. **a** Using the original algorithms that were previously developed using healthy older adults, we were able to achieve high specificity and sensitivity during both the 5 and 10 m TUG. However, the trunk and thigh IMU were prone to false positive (FP) due to sway in the trunk and thigh while participants were sitting in the chair or during pre-posturing by PD participants to gain leverage before standing up. **b** The modified algorithms used the orientation data from the thigh and sacrum modules to calculate the angle of the hip(*θ*_hip_)﻿ to eliminate the FPs detected using the original algorithms. The angle was superimposed on the previous algorithms to eliminate the FPs and increase its accuracy to detect *standing up* and *sitting down*

**Fig. 5:**
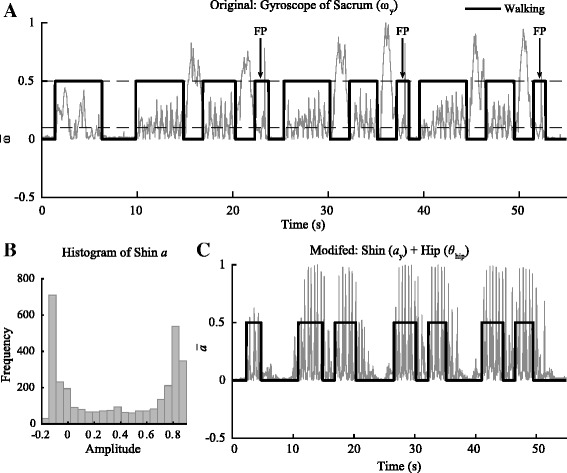
Comparison between the original and modified algorithms during *walking*. The original algorithms used to detect *walking* were based on the gyroscope of the sacrum (ω_y_) and the modified algorithms were based on the acceleration of the shin (*a*_y_) and hip angle (*θ*_hip_). The original algorithms were prone to false positive during *sitting down* (**a**). Furthermore, due to minimal hip movement in the y-direction in some participants who exhibited shuffling gait, detection was more prone to false negative since the signal dropped below the normalized threshold. To remedy this problem, an adaptive threshold was used to set the limit of detection based on the distribution of the amplitude of the signal. This approach adaptively changes the threshold based on the participants; therefore, mitigating the variability among participants (**b**). The acceleration of the shin was adopted to detect walking in PD patients; however, using this IMU alone also yielded many false positives due to extraneous lower limb movements during *sitting down* and *standing up*. To identify true moment of *walking, θ*_hip_ was used to ensure that the participant was standing upright. Therefore, the movement of the shin coupled the upright position of the participant distinguished *walking* from other tasks with 100% accuracy (*n* = 72) (**c**)

Several modifications to the algorithms and IMU selection were made to improve the detection of activities in PD patients (Table 2). These changes were made to enhance the detection algorithms by taking into account the biomechanics and movement strategies adopted by PD patients that were absent in healthy older adults. For example, during *standing up* and *sitting down* (Fig. 4), the angle of the hip (*θ*_hip_) was added to further distinguished these activities from extraneous trunk and hip movements. *θ*_hip_ was calculated using the fused quaternion data of the sacrum and thigh. Furthermore, the band pass filter frequency of the trunk, which was re-optimized using the 10 m TUG data, was reduced from 1.57 Hz to 0.9 Hz to compensate for noise that might have been amplified by the tremors and postural instability. For *walking*, the sacrum IMU was replaced by the shin IMU while a new adaptive thresholding based on the histogram (numbers of bin = 20) of the signal amplitude (*a*_y_, Fig. 5b) was used to set the limit for task detection. This process is similar to Otsu’s thresholding [37] The threshold is defined as:

**Table 2:** Comparison between the modified and original algorithm and IMU selection

Activity	Original methodology	Modified methodology
*Standing up*	• Trunk *a*_z_	• Trunk *a*_z_
	• Thigh *a*_y_	• Thigh *a*_y_
		• *θ*_hip_
*Sitting down*	• Trunk *a*_z_	• Trunk *a*_z_
	• Thigh *a*_y_	• Thigh *a*_y_
	• *f*_cutoff_ = 1.58 Hz	• *θ*_hip_
		• *f*_cutoff_ = 0.9 Hz
*Turning*	• Trunk ω_y_	• Trunk ω_y_
*Walking*	• Sacrum ω_y_	• Shin *a*_y_
	• Normalize thresholding	• *θ*_hip_
		• Adaptive thresholding

1 $$ \mathrm{Threshold}={\mathrm{bin}}_{\max }{\textstyle \hbox{-}}\left({\mathrm{bin}}_{\min }{\textstyle \hbox{-} }{\mathrm{bin}}_{\mathrm{width}}\times \alpha \right) $$

Where bin_max_ and bin_min_ define the maximum and minimum value of the bin of the histogram and α = 0.01. The generalize idea of the methodology is to ensure all viable signals are captured, independent of the strength of the signal, which can often be weak in PD population. With these modifications, the specificity and sensitivity of all the activities were 100% for both tasks (*n* = 432, see Table 3).

**Table 3:** Sensitivity and specificity of activity detection during TUG tasks with original and modified detection algorithms

		Original		Modified	
Activity	TUG	Sens. (%)	Spec. (%)	Sens. (%)	Spec. (%)
*Standing up*	5 m	100	97.3	100	100
	10 m	100	94.7	100	100
*Sitting down*	5 m	100	92.3	100	100
	10 m	100	97.3	100	100
*Turning*	5 m	100	100	100	100
	10 m	100	100	100	100
*Walking*	5 m	88.9	72.2	100	100
	10 m	91.6	87.5	100	100

Modifications in the algorithms and IMU selection used to detect activities in people with PD are presented. The original methodology was developed using a homogenous population of healthy older adults. The accuracy of the algorithms was enhanced in the detection of *standing up*, *sitting down,* and *walking* by concisely redefining the description each activity using the angle of the hip (*θ*_hi*p*_ *)*. The band pass filter frequency was reduced during *sitting down* to compensate for tremor in people with PD and a new adaptive thresholding method was used in the modified algorithm to automatically adapt to each patient and mitigate low signal-to-noise ratio in some participants.

### Segmentation

The segmentation times marked by the algorithms were evaluated using the visual times marked by two independent examiners. A two-way random inter-rater reliability measure was calculated using an intra-class correlation coefficient (ICC). The ICC is scaled from 0 to 1 with the high value indicating excellent reliability. For all transitions segmentation within the TUG, the ICC = 0.99. The differences (mean ± std) of the transition times between the two examiners are summarized in Table 4 (Visual ΔT). On average, the examiners were differed by 175 (±113) *ms* and 156 (±73) *ms* during 10 m and 5 m TUG, respectively.

**Table 4:** The mean and standard deviation of the manual segmentation times marked by two independent examiners

	TUG Transition ΔT					
	Visual ΔT		Org. ΔT	Opt. ΔT	Org. ΔT	Opt. ΔT
	5 m	10 m	5 m		10 m	
*Sit-to-stand*	113 ± 35	235 ± 252	162 ± 67	--	264 ± 239	--
*Stand-to-walk out*	126 ± 84	131 ± 59	345 ± 55	--	309 ± 39	--
*Walk out-to-turn*	184 ± 81	232 ± 83	516 ± 201	--	455 ± 156	--
*Turn-to-walk in*	190 ± 74	128 ± 64	748 ± 277	266 ± 148	856 ± 406	259 ± 177
*Walk in-to-turn*	168 ± 58	204 ± 109	734 ± 143	391 ± 132	469 ± 185	330 ± 188
*Turn-to-stand*	103 ± 76	93 ± 49	958 ± 254	601 ± 225	894 ± 249	612 ± 175>
*Stand-to-sit*	208 ± 106	200 ± 171	197 ± 121	--	188 ± 129	--

**Fig. 6:**
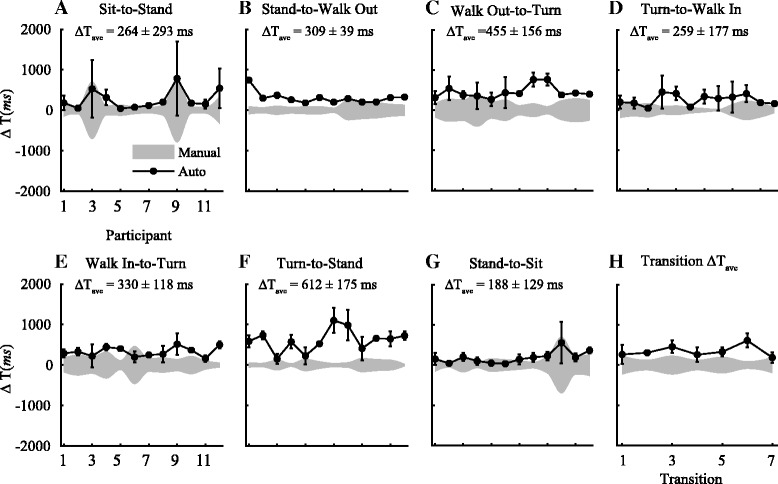
Time difference between visual and auto segmentation time during a 10 m TUG. **a**-**g** ΔT_ave_ defines the time differences between the average segmentation time obtained using the IMUs and the average times marked by two independent examiners (in *milliseconds*) of all twelve participants during a 10 m TUG. **h** Shows the average differences in the transition time across the seven transition points during a TUG. Across the seven transitions, the average difference in the timestamp identified by the IMUs and the visual segmentation was 345 (±157) *ms*

**Fig. 7:**
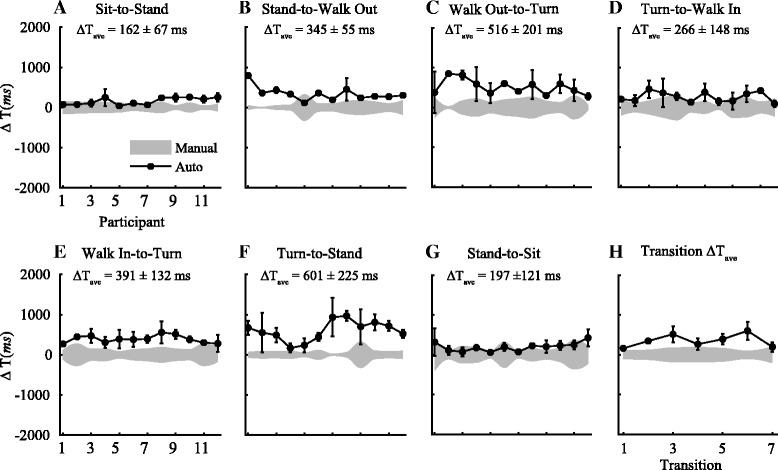
Time difference between visual and auto segmentation time during 5 m TUG. **a**-**g** ΔT_ave_ defines the time differences between the average segmentation time obtained using the IMUs and the average times marked by two independent examiners (in *milliseconds*) of all twelve participants during a 5 m TUG. **h** Shows the average differences in the transition time across the seven transition points during a TUG. Across the seven transitions, the average difference in the timestamp identified by the IMUs and the visual segmentation was 453 (±135) *ms*

Org. ΔT indicates the time differences obtained in the 5 m and 10 m TUG in PD participants using the algorithms that were previously developed based on a population of healthy old adults (Table 4). Across the seven transitions, the average difference between the timestamp obtained using the algorithm and the visual segmentation was 522 (±160) *ms* and 490 (±200) *ms* during the 5 m and 10 m TUG, respectively. The largest difference was during the more dynamics *turn-to-stand* transition for both tasks, while the smallest was during the more static *sit-to-stand* transition. The high cut off frequencies (*f*_cutoff_) were re-optimized during these dynamics transitions using the trials from the 10 m TUG data and applied them to the 5 m TUG (Table 5). The process was previously described [33] in a study using healthy older adults. Using the re-optimized algorithms, the average time difference between the time segmented using the sensors and the examiners across all seven transitions was 453 (±135) *ms* and 345 (±157) *ms* for the 5 m and 10 m TUG, respectively (Table 4, Opt. ΔT, Figs. 6 and 7).

**Table 5:** Original and optimal cutoff frequency for each IMU at different transitions during a TUG

Transition	IMU 1	*f*_cutoff_ (Hz)		IMU 2	*f*_cutoff_ (Hz)	
		Org.	Opt.		Org.	Opt.
*Sit-to-stand*	Trunk *a*_z_	1.57	--	Hip *θ*	0.69	--
*Stand-to-walk out*	Trunk *a*_z_	2.44	--			
*Walk out-to-turn*	Trunk ω_y_	1.32	--	Sacrum ω_y_	0.98	---
*Turn-to-walk in*	Sacrum ω_y_	0.53	3.0			
*Walk in-to-turn*	Trunk ω_y_	1.00	3.0	Sacrum ω_y_	0.59	3.0
*Turn-to-stand*	Trunk ω_y_	0.81	2.0	Sacrum ω_y_	1.00	2.5
*Stand-to-sit*	Trunk *a*_z_	1.02	--			

The high cutoff frequency (*f*_cutoff_) of the band pass filter was re-optimized during more dynamic transitions to increase the accuracy of the segmentation. The optimal range of these cutoff frequencies was between 0.5–3.0 Hz

## Discussion

The aims of this work were to determine the transferability of the detection and segmentation algorithms that were developed based on healthy older adults on people with PD and to adapt the IMU selection and algorithms to the variability in the kinematics patterns of people with PD. While the original algorithms and selected set of IMU from our study in healthy aged individuals [33] performed well during the detection of activities, modification were made to the IMU selection to improve their accuracy in people with PD, especially during *walking*. The same set of IMUs and algorithms were used for segmentation of the activities within the TUG; however, filtering frequencies were re-optimized to improve the segmentation during more dynamic transitions.

Using the original set of IMUs and detection algorithms, we were able to detect the activity such as *standing up*, *sitting down*, and *turning* with an average of 100% sensitivity and 96.7% specificity across the two tasks with only 7 false positives. These false positives were triggered by participants swaying their trunks to gain momentum to stand up from the chair without using their arm or bending their trunk to watch their foot placement before *sitting down*, which were absent in healthy older adults. This in an inherent problem when only one IMU on the trunk is used and only movements of the upper limbs are captured [20, 38, 39]. Even though the number of false positives was minute when compared to the total number of instances detected, it still represents a potential flaw of the automation system under unscripted or semi-scripted task. Superimposing the angle of the hip onto the previous algorithm, we were more successful at distinguishing these movements during *sit-to-stand* and *stand-to-sit* transition. Using additional IMUs, we were able to refine the description of the task; therefore, achieved better accuracy.

While the sacrum IMU was sufficient in detecting *walking* with 100% accuracy in healthy older adults, its performance was significantly reduced in the PD population. This was mainly attributed to the gait differences between healthy and PD population. In some patients, shuffling gaits minimized the angular velocity (*ω*_*y*_) of the hip; therefore it degraded the signal-to-noise ratio in the sacrum IMU dropping the signal below the normalized threshold. Previously, we have used the shin IMU to detect walking in simulated free-living environment [14, 15] with an adaptive thresholding to mitigate the gait variability among participants. However, using the shin IMU alone was not sufficient in isolating *walking* due to extraneous movements that participants with PD might initiate during the TUG. Again, there is a potential for failure when a process is entirely relied on one IMU. However, with a full body set of IMUs, we were afforded the ability to reine the requirements of *walking* using these additional IMUs. These auxiliary details allowed us to remove unscripted movements during the TUG and increase the detection accuracy. Such process can have greater implication during unscripted free-living task in clinical setting and home environment. In *walking*, we used the hip angle in conjunction with the shin acceleration to ensure that the shin movements were actuated when the participants were upright.

Manual segmentation was performed using the visual full body avatar generated from IGS-180 motion capture software and that allowed the examiners to cycle through every frame (60 Hz sampling frequency) to identify the transition point between each activity during the TUG. The transition points identified by the two examiners were within 166 *ms* of each other. However, it took the examiners an average of 30 min to segment one participant performing three trials of a modified TUG. This might be due to the highly subjective nature of manual segmentation and the variability in how participants transition between activities. The automated algorithm based on a system of IMUs could segment these activities just as well as the examiners but at significantly shorter(30 s). Using IMUs and the algorithms that were developed in the previous study, we were able to segment these activities within 500 *ms* of the manual segmentation. However, the more dynamic transitions were less precise relative to manual segmentation. For example, transitions such as *turn-to-walk in*, *walk in-to-turn* and *turn-to-stand* had an average time difference of 813 and 740 *ms* during the 5 m and 10 m TUG, respectively. However, after re-optimizing the high cutoff frequencies in the band pass filter, the time difference was significantly reduced. The optimal range of these cutoff frequencies was between 2-3 Hz. The increase in these optimal cutoff frequencies were due to the over smoothing of the signals that removed viable peaks in the signal that corresponded to the initiation and termination of an activity. The behavior was opposite during activity detection, where signal were over smoothed to remove the noise thus enhancing the visualization of the activity peaks.

Similar studies have demonstrated the viability of using IMUs to detect common activities in daily living. For example, Dijkstra [39] used a single tri-axial accelerometer to measure common physical activity in daily living in controlled environment using patients with mild to moderate PD and were able to generate moderate result with a sensitivity of 60.9-85.4% during *sitting* and *standing*. Jalloul [40] deployed a set of six IMUs on different part of the body to detect common activities such as: *walking* (neck), s*tanding* (wrist) and *sitting* (hip). Using that sensor scheme, Jalloul detected w*alking*, *standing* and *sitting* with 92.4%, 91.8%, 88.6% sensitivity, respectively. It is noted that the algorithm was only tested on 2 patients who were on Levodopa. Salarian [41], using a system of three IMUs was able to detect posture transition such as *sit-to-stand* and *stand-to-sit* in 10 PD patients with a sensitivity of 83.8% (*N* = 272). In classification of common activities such as *walking*, *standing* and *sitting*, we were able to detect these activities with a sensitivity of 98.5%, 97.8%, and 99.8% respectively. Similarly, Zwartjes [42] using a system of 4 IMUs were able to detect these activities with 98% accuracy. As a comparison, our algorithms were able to detect these common activities with 100% accuracy. Furthermore, we also expanded to the detection of turning, which can provide important indication of mobility in people with PD [30, 31, 43–45].

While the goal of the study was not to assess motor performance of patients, it is important to note that patients tested exhibited signs of limited mobility and typical symptomatology associated with PD, such as tremor, bradykinesia, posture instability, and rigidity. In spite of these factors, the algorithms developed here were able to detect the activities in the TUG with 100% accuracy and segmentation was achieved within ~400 *ms* of the ‘gold-standard’ manual segmentation. The time accuracy represents the mean segmentation difference of the four primary activities within a TUG (stand up,sit down, walk and turn) in PD participants. However, when one examines the different activities, the accuracy varies. During sit down and stand up, the time difference with the manual segmentation was only 192  ms (±139), which was only a small percentage of the total time during stand up (1820 ± 160  ms) and sit down (2100 ± 180   ms) [3]. A 400   ms discrepancy during these activities would be significantly large. For PD participants, it took an average of 16 s (16,000   ms) to complete a 5 m TUG; therefore, walking and turning accounted for 75% of the 5 m TUG time (~12 s) and even more in the 10 m TUG. Thus, even the 400 ms difference during walking and turning would not be detrimental to the analysis of the quality of the movement during ‘steady-state’. While the participants did exhibit variable symptomology of early PD, they were able to complete the task with relative ease. Thus, applying the algorithm on a larger PD population with different clinical profiles will be required to further confirm the robustness and reliability of the algorithms. Nevertheless, our results provide a foundation to further investigate the suitability of using IMU to detect and segment daily living activities of people who are diagnosed with PD, regardless of symptomatology.

 Deploying a suit equipped with 17 IMUS is not the most economical system to detect activities within a TUG; however, such system affords us the flexibility to optimize the best set of IMU (see Fig. 1) to detect, segment and analyze the quality of the movement for people with movement disorders. In this study, only 4 IMUs (trunk, sacrum, thigh and shin) were needed to accurately detect and segment the activities within a TUG. We anticipate however that the detection, segmentation and analysis of more complex task in free-living condition might necessitate the need for additional IMUs to distinguish similar activities and capture vital motor behavior associate with movement disorders. This crucial step of activity detection and segmentation will now allow for the development of outcome measures to assess performance and to detect symptomatology within these segments, an important component for the development of a fully-automated system to assess motor performance during a TUG in healthy and diseased populations.

## Conclusions

This study demonstrates the transferability of a detection and segmentation algorithm based on healthy older adults on people with PD. The results show the agility of using a system of IMUs to adapt to mobility-impaired population while maintaining its accuracy in detection and segmentation of common daily living activities. The scope of this study is limited to supervised and scripted activities within the TUG. However, this algorithm is being applied to detect sit down, stand up, walking and turning in more simulated free-living environment in hope of developing a reliable ambulatory system to evaluate motor performance in people with movement disorder in their home environment. Additionally, we continuously work towards improving the use of fusion data using IMUs [46, 47] to aggregate more valuable orientation data that would further streamline the detection and segmentation more complex activities in daily living. Ultimately, the aim of the development of a highly accurate detection and segmentation system is to automate the analysis of task performance in the natural living environment of people who undergo rehabilitation intervention.

## Abbreviations

H&Y: Hoehn and Yahr
IMU: Inertial measurement unit
MOCA: Montreal cognitive assessment test
PD: Parkinson’s disease
TUG: Timed up and go

## References

[1] Center for Disease Control and Prevention (2013). The state of Aging and Health in America 2013.

[2] Havlikova E, Rosenberger J, Nagyova I, Middel B, Dubayova T, Gdovinova Z (2008). Impact of fatigue on quality of life in patients with Parkinson’s disease. Eur J Neurol.

[3] Zampieri C, Salarian A, Carlson-Kuhta P, Nutt JG, Horak FB (2011). Assessing mobility at home in people with early Parkinson’s disease using an instrumented Timed Up and Go test. Parkinsonism Relat Disord.

[4] Boissy P, Briere S, Hamel M, Jog M, Speechley M, Karelis A (2011). Wireless inertial measurement unit with GPS (WIMU-GPS)--wearable monitoring platform for ecological assessment of lifespace and mobility in aging and disease. Conf Proc IEEE Eng Med Biol Soc.

[5] Culhane KM, Lyons GM, Hilton D, Grace PA, Lyons D (2004). Long-term mobility monitoring of older adults using accelerometers in a clinical environment. Clin Rehabil.

[6] Lyons GM, Culhane KM, Hilton D, Grace PA, Lyons D (2005). A description of an accelerometer-based mobility monitoring technique. Med Eng Phys.

[7] Choquette S, Hamel M, Boissy P (2008). Accelerometer-based wireless body area network to estimate intensity of therapy in post-acute rehabilitation. J Neuroeng Rehabil.

[8] Casamassima F, Ferrari A, Milosevic B, Ginis P, Farella E, Rocchi L (2014). A wearable system for gait training in subjects with Parkinson’s disease. Sensors.

[9] Rahimi F, Bee C, Duval C, Boissy P, Edwards R, Jog M. Using Ecological Whole Body Kinematics to Evaluate Effects of Medication Adjustment in Parkinson Disease. Journal of Parkinson’s disease. 2014. doi:10.3233/JPD-140370. 10.3233/JPD-140370 25055960

[10] Moncada-Torres A, Leuenberger K, Gonzenbach R, Luft A, Gassert R (2014). Activity classification based on inertial and barometric pressure sensors at different anatomical locations. Physiol Meas.

[11] Silva J, Monteiro M, Sousa F (2014). Human activity classification with inertial sensors. Stud Health Technol Inform.

[12] Hikihara Y, Tanaka C, Oshima Y, Ohkawara K, Ishikawa-Takata K, Tanaka S (2014). Prediction models discriminating between nonlocomotive and locomotive activities in children using a triaxial accelerometer with a gravity-removal physical activity classification algorithm. PLoS One.

[13] Masse F, Gonzenbach RR, Arami A, Paraschiv-Ionescu A, Luft AR, Aminian K (2015). Improving activity recognition using a wearable barometric pressure sensor in mobility-impaired stroke patients. J Neuroeng Rehabil.

[14] Ayachi F, Nguyen H, Goubault E, Boissy P, Duval C. The Use of Empirical Mode Decomposition-Based Algorithm and Inertial Measurement Units to Auto-Detect Daily Living Activities of Healthy Adults. IEEE Trans Neural Syst Rehabil Eng. 2016. doi:10.1109/TNSRE.2016.2519413. 10.1109/TNSRE.2016.2519413 26829793

[15] Ayachi FS, Nguyen HP, Lavigne-Pelletier C, Goubault E, Boissy P, Duval C (2016). Wavelet-based algorithm for auto-detection of daily living activities of older adults captured by multiple inertial measurement units (IMUs). Physiol Meas.

[16] Del Rosario MB, Wang K, Wang J, Liu Y, Brodie M, Delbaere K (2014). A comparison of activity classification in younger and older cohorts using a smartphone. Physiol Meas.

[17] Lockhart TE, Soangra R, Zhang J, Wu X (2013). Wavelet based automated postural event detection and activity classification with single imu - biomed 2013. Biomed Sci Instrum.

[18] Loh D, Lee TJ, Zihajehzadeh S, Hoskinson R, Park EJ (2015). Fitness activity classification by using multiclass support vector machines on head-worn sensors. Conf Proc IEEE Eng Med Biol Soc.

[19] Najafi B, Aminian K, Loew F, Blanc Y, Robert PA (2002). Measurement of stand-sit and sit-stand transitions using a miniature gyroscope and its application in fall risk evaluation in the elderly. IEEE Trans Bio-Med Eng.

[20] Godfrey A, Bourke AK, Olaighin GM, van de Ven P, Nelson J (2011). Activity classification using a single chest mounted tri-axial accelerometer. Med Eng Phys.

[21] Long X, Yin B, Aarts RM (2009). Single-accelerometer-based daily physical activity classification. Conf Proc IEEE Eng Med Biol Soc.

[22] Zijlstra A, Mancini M, Lindemann U, Chiari L, Zijlstra W (2012). Sit-stand and stand-sit transitions in older adults and patients with Parkinson’s disease: event detection based on motion sensors versus force plates. J Neuroeng Rehabil.

[23] Zijlstra A, Goosen JH, Verheyen CC, Zijlstra W (2008). A body-fixed-sensor based analysis of compensatory trunk movements during unconstrained walking. Gait Posture.

[24] Nemanich ST, Earhart GM (2016). Freezing of gait is associated with increased saccade latency and variability in Parkinson’s disease. Clin Neurophysiol.

[25] Ahlrichs C, Sama A, Lawo M, Cabestany J, Rodriguez-Martin D, Perez-Lopez C (2016). Detecting freezing of gait with a tri-axial accelerometer in Parkinson’s disease patients. Med Biol Eng Comput.

[26] Djuric-Jovicic MD, Jovicic NS, Radovanovic SM, Stankovic ID, Popovic MB, Kostic VS (2014). Automatic identification and classification of freezing of gait episodes in Parkinson’s disease patients. IEEE Trans Neural Syst Rehabil Eng.

[27] Trojaniello D, Ravaschio A, Hausdorff JM, Cereatti A (2015). Comparative assessment of different methods for the estimation of gait temporal parameters using a single inertial sensor: application to elderly, post-stroke, Parkinson’s disease and Huntington’s disease subjects. Gait Posture.

[28] Hubble RP, Naughton GA, Silburn PA, Cole MH (2015). Wearable sensor use for assessing standing balance and walking stability in people with Parkinson’s disease: a systematic review. PLoS One.

[29] Del Din S, Godfrey A, Rochester L. Validation of an accelerometer to quantify a comprehensive battery of gait characteristics in healthy older adults and Parkinson’s disease: toward clinical and at home use. IEEE J Biomed Health Inform. 2015. doi:10.1109/JBHI.2015.2419317. 10.1109/JBHI.2015.2419317 25850097

[30] Mellone S, Mancini M, King LA, Horak FB, Chiari L (2016). The quality of turning in Parkinson’s disease: a compensatory strategy to prevent postural instability?. J Neuroeng Rehabil.

[31] Yang WC, Hsu WL, Wu RM, Lu TW, Lin KH (2016). Motion analysis of axial rotation and gait stability during turning in people with Parkinson’s disease. Gait Posture.

[32] Daneault JFC, B; Sadikot, A.F.; Panisset, M; Duval, C. Drug-induced dyskinesia in Parkinson’s disease. Should success in clinical management be a function of improvement of motor repertoire rather than amplitude of dyskinesia? BMC Med. 2013;Accepted. 10.1186/1741-7015-11-76 PMC3751666 23514355

[33] Nguyen HP, Ayachi F, Lavigne-Pelletier C, Blamoutier M, Rahimi F, Boissy P (2015). Auto detection and segmentation of physical activities during a Timed-Up-and-Go (TUG) task in healthy older adults using multiple inertial sensors. J Neuroeng Rehabil.

[34] Nasreddine ZS, Phillips NA, Bedirian V, Charbonneau S, Whitehead V, Collin I (2005). The Montreal Cognitive Assessment, MoCA: a brief screening tool for mild cognitive impairment. J Am Geriatr Soc.

[35] Hoehn MM, Yahr MD (1967). Parkinsonism: onset, progression and mortality. Neurology.

[36] Rogers S, et al. A first course in machine learning: Machine learning & pattern recognition. 2nd ed. Machine learning & pattern recognition. 2015, Boca Raton, FL: Chapman and Hall/CRC Press.

[37] Otsu N (1975). A threshold selection method from gray-level histograms. Automatica.

[38] van Lummel RC, Walgaard S, Hobert MA, Maetzler W, van Dieen JH, Galindo-Garre F (2016). Intra-Rater, inter-rater and test-retest reliability of an instrumented timed up and go (iTUG) Test in patients with Parkinson’s disease. PLoS One.

[39] Dijkstra B, Kamsma YP, Zijlstra W (2010). Detection of gait and postures using a miniaturized triaxial accelerometer-based system: accuracy in patients with mild to moderate Parkinson’s disease. Arch Phys Med Rehabil.

[40] Jalloul N, Poree F, Viardot G, L’Hostis P, Carrault G (2015). Detection of Levodopa Induced Dyskinesia in Parkinson’s Disease patients based on activity classification. Conf Proc IEEE Eng Med Biol Soc.

[41] Salarian A, Russmann H, Vingerhoets FJ, Burkhard PR, Aminian K (2007). Ambulatory monitoring of physical activities in patients with Parkinson’s disease. IEEE Trans Bio-Med Eng.

[42] Zwartjes DG, Heida T, van Vugt JP, Geelen JA, Veltink PH. Ambulatory monitoring of activities and motor symptoms in Parkinson’s disease. IEEE transactions on bio-medical engineering. 2010;57(11). doi:10.1109/TBME.2010.2049573. 10.1109/TBME.2010.2049573 20460198

[43] El-Gohary M, Pearson S, McNames J, Mancini M, Horak F, Mellone S (2013). Continuous monitoring of turning in patients with movement disability. Sensors.

[44] Stack EL, Ashburn AM, Jupp KE (2006). Strategies used by people with Parkinson’s disease who report difficulty turning. Parkinsonism Relat Disord.

[45] Cheng FY, Yang YR, Wang CJ, Wu YR, Cheng SJ, Wang HC (2014). Factors influencing turning and its relationship with falls in individuals with Parkinson’s disease. PLoS One.

[46] Lebel K, Boissy P, Hamel M, Duval C (2013). Inertial measures of motion for clinical biomechanics: comparative assessment of accuracy under controlled conditions - effect of velocity. PLoS One.

[47] Lebel K, Boissy P, Hamel M, Duval C (2015). Inertial measures of motion for clinical biomechanics: comparative assessment of accuracy under controlled conditions - changes in accuracy over time. PLoS One.

